# The Role of POPDC Proteins in Cardiac Pacemaking and Conduction

**DOI:** 10.3390/jcdd8120160

**Published:** 2021-11-23

**Authors:** Lena Gruscheski, Thomas Brand

**Affiliations:** National Heart and Lung Institute, Imperial College London, London W12 0NN, UK; l.gruscheski@imperial.ac.uk

**Keywords:** cyclic nucleotide signaling, cyclic nucleotide binding, sinus bradycardia, atrioventricular block, striated muscle, limb-girdle muscular dystrophy

## Abstract

The Popeye domain-containing (POPDC) gene family, consisting of *Popdc1* (also known as *Bves*)*, Popdc2*, and *Popdc3*, encodes transmembrane proteins abundantly expressed in striated muscle. POPDC proteins have recently been identified as cAMP effector proteins and have been proposed to be part of the protein network involved in cAMP signaling. However, their exact biochemical activity is presently poorly understood. Loss-of-function mutations in animal models causes abnormalities in skeletal muscle regeneration, conduction, and heart rate adaptation after stress. Likewise, patients carrying missense or nonsense mutations in POPDC genes have been associated with cardiac arrhythmias and limb-girdle muscular dystrophy. In this review, we introduce the POPDC protein family, and describe their structure function, and role in cAMP signaling. Furthermore, the pathological phenotypes observed in zebrafish and mouse models and the clinical and molecular pathologies in patients carrying POPDC mutations are described.

## 1. Introduction

The heart consists of two different kinds of myocytes, i.e., the working or force-producing cardiac myocytes found in the atrial and ventricular chambers, and the nodal and conducting myocytes forming the cardiac conduction system (CCS). The CCS spontaneously generates and propagates electrical activity to trigger the synchronous and consecutive contraction of the atrial and ventricular chambers [1,2]. Atrial and ventricular myocytes are characterized by the presence of stable resting membrane potential, while CCS myocytes are characterized by spontaneous diastolic depolarization. During each cardiac cycle, myocytes of the sinoatrial node (SAN) are the fastest to depolarize and first to generate action potential [1]. From the SAN, the electrical activation spreads to the atrial myocardium and subsequently to the ventricular chambers via the atrioventricular node (AVN). Synchronous electrical activation of the ventricular chambers is achieved by rapid propagation of the action potential from the base to the apex via the His bundle and bundle branches, which are running along the ventricular septum [2]. The distal part of the ventricular CCS is made up of the Purkinje fiber (PF) network. Like the His bundle and bundle branches, PF have fast conduction properties, allowing an electrical activation of the ventricles from the apex to base, which is essential for efficient blood pumping of the ventricular chambers. A fascinating aspect of the orchestration of electrical activity in the mammalian heart is its adaptability i.e., the heart is able to beat faster or slower depending on the physiological demand. Communication with the body is mediated by the autonomous nervous system through the release of neurotransmitters such as norepinephrine and acetylcholine, which trigger signaling cascades at the postsynaptic membrane of cardiac myocytes [3]. This review deals with the Popeye domain-containing (POPDC) proteins, a family which is strongly expressed in the heart and important for cardiac pacemaking and conduction. We first review the genomic organization of the POPDC genes and the structure and function of the encoded proteins. POPDC proteins are novel mediators of cyclic nucleotide signaling and we briefly review this signaling pathway and then discuss the role of POPDC proteins in this context. Loss of POPDC genes in model organisms are associated with cardiac arrhythmia and we review the current state of knowledge. Apart from its role in electrical activation of the heart, there is also evidence for other plasma membrane functions in striated muscle and these are briefly discussed. A growing number of patients carrying mutations in one of the three POPDC genes have been identified. The mutations are associated with cardiac arrhythmia and muscular dystrophy and we outline the disease association of POPDC genes. Finally, we give an outlook and identify areas where novel insight will be required in the future.

## 2. Genomic Organization and Gene Regulation of POPDC Genes 

The Popeye domain-containing (POPDC) gene family was first discovered when screening cDNA libraries for novel genes with cardiac-restricted expression [4,5]. Three genes were identified, namely, *POPDC1* (also known as blood vessel epicardial substance (*BVES*), *POPDC2*, and *POPDC3* [4,5]. In man, POPDC genes are present on two chromosomal loci; *POPDC1* and *POPDC3* are organized in tandem on chromosome 6q21, and *POPDC2* is localized to chromosome 3q13.33 [4]. The tandem configuration of *POPDC1* and *POPDC3* is preserved down the evolutionary tree to lower chordates, which suggests importance for coordinated gene regulation [6]. Indeed, studies of gene expression in failing hearts have shown a co-regulation of both *POPDC1* and *POPDC3,* and a concerted reduction in gene expression in a large proportion of heart failure patients, while *POPDC2* apparently is independently regulated [7]. Similar results were recently obtained in patients with gastric cancer, which showed a strong correlation between a reduction in the transcript levels of *POPDC1* and *POPDC3* and the transition from premalignant to a malignant state, while *POPDC2* expression levels were independent of the cancer state [8]. *POPDC2* is only found in vertebrates and is thought to have arisen by gene duplication from *POPDC3*, showing approximately 50% sequence homology at the protein level [4]. In comparison, sequence homology at the the protein level of either POPDC2 or POPDC3 to POPDC1 is only about 25%. Therefore, it can be hypothesized that *POPDC1* was the primordial gene and *POPDC2* and *POPDC3* arose through two separate gene duplication events: the first duplication producing the tandem organization of *POPDC1*–*POPDC3* probably occurred during early chordate evolution [6]. The second gene duplication of *POPDC3* to produce *POPDC2* probably happened during early vertebrate evolution given that in teleosts, *POPDC2* is present, whereas in lower chordates such as tunicates, only *POPDC1* and *POPDC3* are found. 

Expression of one or more POPDC isoforms is found in a variety of smooth muscle-containing organs (bladder, uterus, gastrointestinal tract, and the lung), neurons of the central and autonomic nervous system, and in several types of epithelial cells (epidermal cells, stomach, and cornea) [9,10,11,12,13]. However, by means of immunohistochemistry, beta-galactosidase staining of knock-in reporter genes, RT-PCR, and in situ hybridization, the highest expression of POPDC genes has been found in striated muscle cells (skeletal muscle and heart) [4,9,12,14]. Of the three POPDC genes, *POPDC1* is more widely expressed and can be found at relatively high levels in both skeletal and cardiac muscle cells, while *POPDC2* is predominantly found in the heart and *POPDC3* mainly in skeletal muscle. 

Little is known about the signaling pathways and DNA regulatory elements controlling the expression of POPDC genes. Expression of *Popdc1* in muscle cells may be under the control of PAX3 during mouse embryogenesis and in alveolar rhabdomyosarcoma [15]. In human hepatocellular carcinoma (HCC), netrin-1, a laminin-related neuronal guidance molecule, negatively regulates *POPDC1* expression through AKT activation [16]. *POPDC1* and *POPDC3* promoter hypermethylation and histone modifications have been suggested as major mechanisms by which both genes are downregulated in gastric and colon cancer [17,18]. 

Epidermal growth factor (EGF) signaling appears to negatively regulate *POPDC1* expression in breast cancer and adenocarcinoma cells [19]. Consistent with these observations in cancer cells, during oogenesis in *Drosophila*, the EGF-like ligand Gurken was found to suppress *popdc1* expression in dorsal follicle cells [20]. Negative regulation of *POPDC* gene expression by the addition of serum has been described in neonatal rat cardiac myocytes and likely also involves EGF since it can be blocked by the EGF receptor antagonist tyrphostin [21]. 

*Popdc2* is strongly expressed in cardiac muscle cells and the promoters contain binding sites for Meis homeobox protein 1 (Meis1) and NK homeobox protein 2.5 (Nkx2.5) [22]. Meis1 was shown to occupy the *Popdc2* promoter during anterior heart field formation and Nkx2.5 was bound to the *Popdc2* promoter in differentiated cardiac myocytes. The strong cardiac myocyte-specific expression of POPDC2 was recently exploited to purify cardiac myocytes from human embryonic stem cell cultures [23]. 

## 3. The POPDC Protein Family

At the protein level, POPDC1 and POPDC2 are differentially expressed in the heart. POPDC1 is expressed at higher levels in the atria than in the ventricles, whereas POPDC2 is expressed evenly across the whole heart. Importantly, both proteins are expressed at elevated levels in the CCS. 

**Figure 1:**
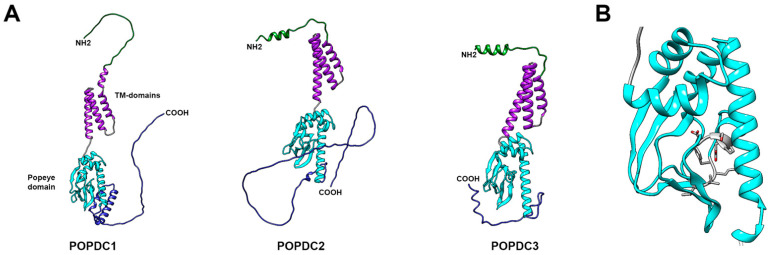
Structural modeling of human POPDC proteins. The structural models were generated by AlphaFold [27]. (**A**) Structural comparison of POPDC1, POPDC2 and POPDC3. Each POPDC protein consists of four distinct regions: the extracellular amino terminus (green), three transmembrane domains (purple), the highly conserved Popeye domain (light blue), and the carboxy terminus, which is predicted to be disordered (dark blue). (**B**) Structural model of the Popeye domain of POPDC1, which functions as a cAMP-binding domain. Like other cAMP-binding domains, it is composed of three alpha helices and an eight-stranded, antiparallel beta-barrel structure. Two ultra-conserved motifs, DSPE and FQVT (residues are labeled in grey), are believed to form an atypical phosphate-binding cassette (PBC) [14,28].

In the adult heart, POPDC protein expression is confined to cardiac myocytes and absent from non-muscle cells [12]. In cardiac myocytes, POPDC proteins are localized to the lateral membrane, intercalated disk, t-tubules, caveolae, and costameres [24]. POPDC proteins are medium-sized transmembrane proteins (POPDC1: 360aa, POPDC2: 364aa, POPDC3: 291aa) and consist of four distinct regions: amino terminus, three transmembrane domains, highly conserved cytosolic Popeye domain, and a carboxyl terminus (Figure 1). The extracellular amino terminus consists of 27–39 residues and is probably too short to be directly involved in cell–cell adhesion as originally proposed by Wada et al. [25]. However, two conserved and functional N-glycosylation sites were mapped to the amino terminus of POPDC1, while a single site is present in the amino terminus of POPDC2 and POPDC3 [10,26]. 

The level of glycosylation is extensive and tissue-specific, leading to an increase in the MW of POPDC1 from its predicted value of 41kDa to 58kDa in the heart, and to 70 kDa in skeletal muscle and the brain when measured by Western blot analysis [26,29]. It is currently unclear whether the extensive glycosylation of POPDC proteins is important. In general, glycosylation determines protein stability. Abnormal glycosylation and glycan branching patterns have been implicated in aberrant cell–cell adhesion and promotion of cell invasion and metastasis [30]. Furthermore, in skeletal muscle, O-linked mannosylation of α-dystroglycan is essential for linking the dystrophin-associated glycoprotein complex to laminin [31]. Thus, it is possible that the tissue-specific glycosylation of POPDC1 proteins may be of functional importance and deserves to be further studied.

The hydrophobic transmembrane region consists of three alpha helices by which the protein is anchored to the plasma membrane. POPDC1 proteins form homodimers that are stabilized by an intermolecular disulfide bridge [26]. Utilizing a series of experiments such as carboxyterminal deletion analysis, site-directed mutagenesis, and peptide-mapping, a dimerization motif was mapped to two conserved lysine residues of the carboxyl terminus of the Popeye domain [32]. However, it is noteworthy that others have suggested alternative sequences to be essential for protein homodimerization as POPDC1 proteins were still able to form homodimers even after the proposed dimerization motif was truncated [33]. These findings suggest that multiple sites in the protein are probably involved in formation of stable homodimers and may be able to compensate for each other. At present, it is unknown whether heterodimer formation also occurs amongst POPDC isoforms. However, patients carrying mutations in *POPDC1* often display a loss of membrane localization of both POPDC1 and POPDC2, which suggests that the two proteins might be undergoing heteromeric complex formation [29]. 

The cytoplasmic region of POPDC proteins contains the 150 amino acid-long, highly conserved Popeye domain, which functions as a 3′,5′-cyclic adenosine monophosphate (cAMP)-binding site [14] (Figure 1B). Sequence homology of the Popeye domain to other cAMP-binding domains is limited, while sequence conservation of the Popeye domain reaches up to 60% between POPDC isoforms [4]. Aside from POPDC proteins in different species, the protein with highest sequence similarity to the Popeye domain with 25% sequence identity and 60% sequence similarity is the bacterial catabolite activator protein (CAP), also known as cAMP receptor protein (CRP) [14]. CRP functions as a transcription factor that controls the expression of enzymes involved in carbohydrate metabolism. Binding of cAMP increases the affinity of CRP for its target DNA sequence, therefore functioning as its effector [34]. Work by Schindler et al., 2012 and others demonstrated a localization of POPDC1 to the nuclear envelope and nucleoplasm in several cell types such as activated and cycling satellite cells [35,36]. Nuclear localization appears to be regulated by differentiation and a loss of the nucleoplasmic expression domain was observed in myotubes. It can be speculated that POPDC proteins might have evolved from a transcriptional regulator, gaining a transmembrane domain. Alternatively, POPDC proteins could, in addition to their role at the plasma membrane, also have nuclear functions such as regulating gene expression in a cAMP-dependent manner. 

The carboxyl terminus of POPDC proteins is variable in length and isoform-specific. It is subject to alternative splicing in POPDC1 and POPDC2, predicted to be structurally disordered, and to contain regions of low complexity [37] (Figure 1). Regions of low complexity have the flexibility to attain a specific secondary structure upon binding of an interaction partner. These disorganized regions are often found in so-called hub proteins that serve at the center of protein–protein networks due to their ability to interact with a large number of protein-interaction partners [38,39]. Attributed to its carboxyterminal regions of low complexity, POPDC1 has recently been identified as a hub protein in the context of atrial fibrillation [40]. Proteomic analysis revealed that all three POPDC proteins have β-adrenergic receptor (βAR)-dependent phosphorylation sites within their carboxyl terminus [41]. Therefore, β-adrenergic signaling might modulate the biological activity of POPDC proteins not only through cAMP binding, but possibly also through phosphorylation, which could also have an impact on protein–protein interaction [29,42,43,44,45,46]. 

## 4. Elements of the cAMP Signaling Pathway

The cyclic nucleotide cAMP, which was first discovered by Earl W. Sutherland [47], is one of the most important second messengers found in eucaryotic cells and is involved in many important signaling pathways to mediate a plethora of cellular responses [48,49]. In the heart, it has been shown, for example, that cAMP accumulates after β-adrenoceptor (βAR) stimulation and translates into an increased contractility, beating frequency, rate of relaxation, excitability, and conductivity [50]. Both βAR subtypes, β_1_ and β_2_, initiate a rapid raise in cAMP levels. However, the resulting downstream cellular responses are quite different: sustained β_1_AR activation causes myocyte apoptosis and β_2_AR activation is cardioprotective [51]. Nikolaev and colleagues argue that the difference in the observed physiological responses is due to a differential distribution of β_1_AR and β_2_AR receptors in cardiac myocytes. β_1_AR are evenly distributed in the sarcolemma, whereas β_2_AR are mainly confined to transverse tubules (t-tubules) [52]. T-tubules are extensions of the cell membrane that penetrate the center of striated muscle cells. They are essential for the rapid propagation of the action potential and an efficient and synchronous excitation–contraction coupling [53]. In line with the subtype-specific subcellular localization, cAMP that is produced in response to β_1_AR activation propagates through the entire cell, whereas the β_2_AR response is confined to t-tubules [54]. Failure to compartmentalize cAMP after β_2_AR activation results in an aberrant signal propagation and is thought to be associated with heart failure [52]. These findings are in line with the recent school of thought that cAMP diffusion is spatiotemporally restricted and confined to nanodomains, where cAMP is locally generated and degraded [55,56]. A restrictive regulation of cAMP activity to discrete sub-cellular compartments therefore provides a mechanism by which cAMP can mediate many different cell type- and ligand-specific responses. 

Compartmentalization of cAMP signaling requires the formation of different protein complexes. Signal propagation is initiated when a ligand binds to G-protein-coupled receptors (GPCR) such as β_1_- or β_2_AR, which in cardiac myocytes are differentially distributed across the plasma membrane, spatially restricting the cAMP response. These GPCRs undergo confirmational change in response to ligand binding, triggering the activation of Gα_s_. Gα_s_ stimulates adenylyl cyclase (AC), which catalyze the synthesis of cAMP from adenosine triphosphate (ATP). In contrast, inhibitory Gα_i_ subunits block AC activation, thereby limiting cyclic AMP production [57]. 

There are 10 AC isoforms, of which nine are membrane bound (AC1–AC9) and one that is soluble (sAC) [58]. Five isoforms, AC1, AC5, AC6, AC8, and AC9, are mainly found in the heart. AC1 and AC8 are stimulated by Ca^2+^ through calmodulin and inhibited by Gβγ subunits [59] and are expressed at high levels alongside AC5 and AC6 in the SAN of the rabbit and guinea pig [60,61]. Transgenic mice overexpressing AC8 show an increased heart rate and a reduced heart rate variability [62]. Null mutants for either *Adcy1* or *Adcy8* display a normal heart rate, which may be due to a functional redundancy of these two isoforms [63]. AC5 and AC6 are the most abundantly expressed isoforms found in the rodent heart. Both proteins are activated through similar regulatory pathways and are inhibited by Ca^2+^, but they differ in their subcellular localization and function [64]. Studies in mice have shown that AC5 is largely associated with stress responses and AC6 is necessary for Ca^2+^ handling and contractility [58]. Finally, AC9 is a unique AC isoform which has a low sensitivity to the small molecule AC activator forskolin (FSK) [59]. In the heart, AC9 forms a complex with the A kinase anchor protein (AKAP) Yotatio, potassium voltage-gated channel subfamily Q member 1 (KCNQ1), and protein phosphatase 1 (PP1) [65]. AC9 is essential for cardiac pacemaking as null mutants for *Adcy9* present sinus bradycardia and a diastolic dysfunction with preserved ejection fraction [65]. The different AC isoforms show differential subcellular localization. AC5, AC6, and AC8 are expressed in membrane rafts, where they respond to local changes in Ca^2+^. In the heart, AC6 is concentrated in caveolae, which are caveolin-rich domains where its activity can selectively be modulated by β_2_AR activation [59].

Phosphodiesterases (PDEs) are a large superfamily of enzymes that catalyze the degradation of cAMP and/or 3′,5′-cyclic guanosine monophosphate (cGMP) to AMP and/or GMP, respectively, positioning them as highly important regulators of cyclic nucleotide diffusion and signaling. In the context of the heart, it has been shown that at least five isoforms, PDE1–PDE5 are expressed in the ventricles, of which PDE3 and PDE4 are the major isoforms. In the mouse heart, 60% of all PDE activity is attributed to PDE4, with PDE3 activity accounting to 30% [66]. On the other hand, in the human heart, PDE3 is the predominant PDE isoform, and, in combination with PDE1 and PDE2, they cover 90% of all PDE activity [67,68]. Inhibition of PDE3 activity increased basal SAN beating frequency in the mouse or human heart. However, PDE3 or PDE4 blockers did not affect cardiac pacemaking after βAR stimulation [69]. Therefore, it has been concluded that although PDE3 and PDE4 are important for basal pacemaker activity, they are probably not involved in upregulation of SAN function in response to adrenergic stimulation. 

It is noteworthy that effective cAMP compartmentalization results from effective targeting and tethering of a distinct and unique combination of scaffolding, effector, and regulatory proteins into so-called signalosomes. PDE integration into these signaling complexes is isoform-specific and the relative abundance of a particular PDE isoform is therefore not essentially reflective of its overall importance for cellular function. This has been elegantly reviewed recently by Tibbo and Baillie, describing the functional role of PDE4B in the brain [70].

## 5. cAMP Effector Proteins

Elevated cAMP levels can activate a wide range of cellular processes through specific interaction with effector proteins that contain a cyclic nucleotide-binding domain (CNBD). There are four cAMP effectors, namely, protein kinase A (PKA), exchange protein activated by cyclic AMP (EPAC1 and -2), cyclic nucleotide-gated ion channels (CNG), and POPDC proteins.

PKA is the first effector protein that was discovered and is currently also the best characterized. It is made up of two regulatory subunits, of which there are two classes: RI and RII, and two catalytic subunits of which there are three isoforms called Cα, Cβ, and Cγ [71]. Under physiological conditions, cAMP binds to the regulatory subunits by which the catalytic subunits are getting activated. Each isoform is encoded by a unique gene and preferentially expressed in different cells and tissues. PKA signaling is mediated through the phosphorylation of a plethora of intracellular proteins, including proteins regulating energy metabolism, cardiac pacemaking, and excitation–contraction coupling [71]. PKA is targeted to various subcellular domains by scaffolding proteins called A kinase anchoring proteins (AKAPs) [72].

AKAPs are a structurally diverse group of proteins with the unifying feature being the presence of a 14–18 amino acid-long protein kinase-binding domain, which forms an amphipathic helix through which AKAP proteins interact with the N-terminal D/D domain of the regulatory subunit dimer of PKA [73]. 

Additionally to PKA targeting, AKAP proteins function as scaffolding proteins upon which signaling complexes can assemble for cAMP and also other signaling pathways [64]. In the heart, the importance of AKAPs has been demonstrated in the case of calcium-induced calcium release (AKAP18α, AKAP18γ, AKAP79), repolarization (Yotatio, D-AKAP2), and stress response (AKAP-Lbc, mAKAPβ, D-AKAP1), to name a few [74].

There are two EPAC isoforms, EPAC1, which has one and EPAC2, which has two cAMP-binding domains. They function principally as guanine nucleotide exchange factors via their GEF domain to stimulate Ras-like GTPases, Rap1 and Rap2. In cardiac myocytes, both EPAC isoforms are expressed albeit at different subcellular localizations and thought to modulate Ca^2+^ homeostasis and hypertrophy [75]. EPAC1 is predominantly localized perinuclearly and EPAC2 is mostly present at the t-tubules [76]. EPAC1 was also shown to be present in mitochondria and its loss is associated with a reduced infarct size as well as reduced cardiomyocyte apoptosis following myocardial ischemia/reperfusion injury [77]. Additionally, EPAC was implicated in cardiac hypertrophy after chronic activation [78] and was shown to have pro-arrhythmic effects by decreasing potassium currents in cardiac myocytes [79]. 

CNG channels are found in a variety of tissues such as the kidney, brain, and heart. One class of CNG present in the heart and brain are called hyperpolarization-activated cyclic nucleotide-gated (HCN) channels. They are found in parts of the ventricular conduction system but are predominantly expressed in the SAN and the AVN, where three isoforms (HCN1, HCN2, and HCN4) of the four HCN channels are expressed [80]. A unique property of HCN channels is their opening by hyperpolarization and gating, which is facilitated by binding of cAMP to a CNBD in the C-terminus of the channel. Different HCN channels present variable characteristics such as kinetics, voltage dependence, and affinity to cAMP [81]. For example, HCN4 has the highest cAMP affinity whilst HCN1 shows only low cAMP sensitivity. Furthermore, HCN4 channels, compared to HCN1 channels, show the slowest activation and deactivation kinetics, and open at more negative potentials. HCN4 channels are primarily responsible for I_f_, an important pacemaker current, which is activated at hyperpolarized membrane potential and drives slow diastolic depolarization (SDD), which contributes to the spontaneous pacemaker cell activity [82]. 

HCN channels, alongside several other electrogenic proteins, contribute to the automaticity of SAN pacemaker cells and are collectively termed the membrane clock [83]. However, SAN pacemaking also involves intracellular Ca^2+^ cycling, and proteins involved in Ca^2+^ release and re-uptake are termed the Ca^2+^ clock [84]. Traditionally, both clocks are thought to be spatially and functionally separated, however, recent findings have shown that pacemaker cells are unable to generate a spontaneous action potential when the two clocks become experimentally uncoupled. Thus, the proper timing of membrane and intracellular events is of exquisite importance in order to produce a coupled-clock system, which is required to drive automaticity of SAN pacemaker cells and to generate a stable heart rhythm (intrinsic entrainment) [85].

The activity of many proteins associated with the membrane and calcium clock are modulated by the autonomous nervous system (ANS) (neuronal entrainment). HCN4, the major HCN channel isoform in the SAN, has been proposed to act as the main effector of ANS-mediated heart rate (HR) regulation (chronotropic effect). HCN4-channel gating is affected by changes in cytosolic cAMP levels, which in turn are tightly controlled by the ANS: activation of the sympathetic nervous system leads to an increase in cAMP levels, increasing the I_f_ current, which results in accelerated SDD, producing a higher firing rate of SAN cells, and ultimately increasing the HR. Conversely, activation of the parasympathetic nervous system reduces cAMP levels, which leads to a slowdown in HR.

Work with mouse knockout models revealed that null mutants for *Hcn1* and *Hcn4* show defective cardiac pacemaking [86,87]. *Hcn1* null mutants present postnatal sinus bradycardia and HCN4 null mutants show embryonic lethality due to severe bradycardia. Additionally, it was observed that the application of ivabradine, which antagonizes I_f_ by binding to HCN channels in their closed state, reduced the heart rate in heart failure patients [88]. Collectively, these data are compatible with the hypothesis that HCN4-channel activity mediates the chronotropic effect. However, other work does not support this conclusion. HCN4-channel gating kinetics are too slow to mediate ANS-triggered changes in the firing rate of pacemaker cells and the successive change in sinus rhythm [89]. Furthermore, adult mice with a SAN-specific loss of *Hcn4* retain their ability to respond to sympathetic stimulation, despite progressively developing bradycardia and AV block [90]. In addition, Kozasa et al. (2018) showed that overexpression of *Hcn4* does not induce tachycardia, however, conditional knockdown of *Hcn4* results in bradycardia, sinus arrhythmia, and enhanced parasympathetic responses to cervical vagus nerve stimulation [91]. Thus, these experiments suggest that instead of mediating the chronotropic response of the SAN, HCN4 channels’ main role is to act as a depolarization reserve, which is important for attenuating parasympathetic responses by stabilizing spontaneous firing of the SAN [92]. HCN1 channels are important during the phasic entrainment process. They stabilize the membrane potential in the subthreshold diastolic voltage range and attenuate any interfering influences from neighboring pacemaker and atrial cells during action potential firing, thereby suppressing heart rate fluctuations [93,94]. Thus, both HCN1 and HCN4 are involved in ensuring the robustness of cardiac pacemaking but they are probably not essential for heart rate adaptation after adrenergic receptor stimulation. 

**Figure 2:**
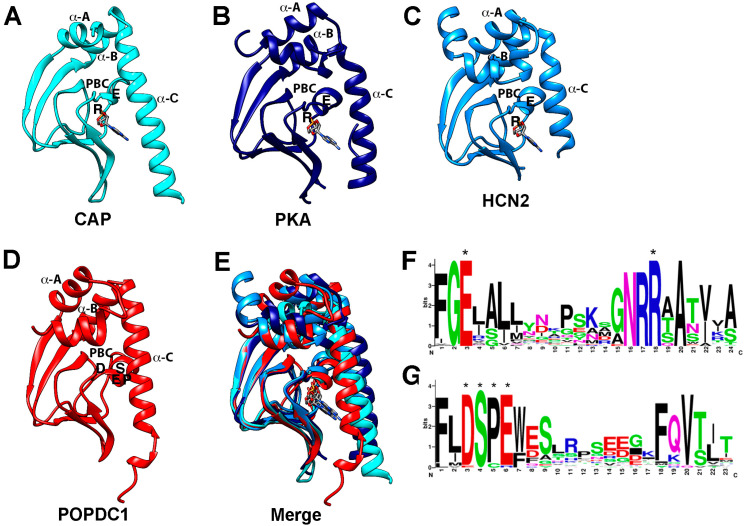
Structure of the CNBD and sequence of the PBC of cAMP effector proteins. (**A**–**C**) Crystal structure of the CNBDs of (**A**) CAP protein of E. coli (PDB: 1CGP), (**B**) type II beta regulatory subunit of PKA of Rattus norvegicus (PDB: 1CX4), (**C**) HCN2 of Mus musculus (PDB:1Q43). Note that in each case the cAMP bound structure is shown. (**D**) Alpha-Fold prediction of the CNBD of POPDC1 of Homo sapiens. In each protein model, the α-A, α-B (hinge), and α-C (lid) helices are labeled. Likewise, the conserved R and E residues in the PBC of the canonical CNBDs and the DSPE motif in the PBC of POPDC1 are indicated. (**E**) Overlay of the protein structures shown in (**A**–**D**) revealing a similar tertiary structure of the CNBDs. (**F**,**G**) Comparison of the PBC of (**F**) canonical cyclic nucleotide monophosphate (cNMP)-binding proteins and (**G**) POPDC proteins. In the PBC of canonical cAMP-binding proteins, two highly conserved sequence motifs (FGE and NRR) are present and the E and R residues (labeled by asterisks, also depicted in the individual crystal structures shown in (**A**–**C**)) bind to the phosphate group of the cyclic nucleotide. In contrast, the PBC of POPDC proteins contains the ultra-conserved sequence motifs DSPE (labeled by asterisks) and FQVT and modeling and mutagenesis suggest direct involvement in cAMP binding. The canonical PBC strongly diverges from the one present in POPDC proteins. Sequence data utilized for the generation of the logo for the Popeye domain were retrieved from the seed-sequence collection of the PFAM entry of the Popeye domain (PF04831) and for the PBC of canonical cAMP-binding proteins. The logo was downloaded from Prosite (PS00889; CNMP_BINDING_2), https://prosite.expasy.org/PDOC00691 accessed on 22 November 2021).

POPDC proteins are the most recent addition to the group of cAMP effector proteins. All three family members contain the highly conserved Popeye domain, which is present from hydra to men [4]. To date, producing an empirical or crystal structure of the Popeye domain or indeed of the whole POPDC protein has not been accomplished. However, secondary structure predictions of the Popeye domain revealed similarities to the canonical CNBD as found in PKA and EPAC [14]. This led to the development of a homology-based structural model of the Popeye domain, with the structures of CRP and PKA RII as templates [14] (Figure 2). A classical CNBD consists of a highly conserved phosphate-binding cassette (PBC), a helical bundle (α-A) at the N-terminus, and hinge (α-B) and lid regions (α-C) at the C-terminus, collectively often described as a jolly-roll β-barrel fold structure [95]. The PBC, an essential part of the CNBD, contains a short loop and an α-helix between two β-sheets, where direct interaction with cAMP occurs [96]. Binding of cAMP induces re-orientation of the PBC that causes conformational change at the hinge, lid, and subsequently the helical bundle regions [95]. The bound conformation is stabilized through the interaction of the lid with the adenosine base of the cAMP molecule. Downstream events after cAMP binding are initiated through the positioning of the lid region [95]. For the Popeye domain, the homology model predicted the presence of an α-helical region with an adjacent β-sheet, somewhat resembling the classical CNBD [14] (Figure 2). This putative CNBD contains two ultra-conserved motifs, DSPE and FQVT, which are predicted to form an atypical PBC directly involved in cAMP binding (14). The jelly-roll β-barrel fold structure is not exclusive to proteins that bind cyclic nucleotides and is also found in many proteins that bind other ligands [97]. However, a series of experiments provided strong empirical evidence that the Popeye domain functions as a cAMP-binding domain [14]. A radioligand binding assay using the recombinant Popeye domain of POPDC1 and a bimolecular Förster-resonance energy transfer (FRET) assay, based on the protein–protein interaction of POPDC1 and the two-pore potassium channel TREK-1, established that POPDC proteins are able to bind cAMP with an affinity similar to that of PKA [14]. At high concentrations, binding of cGMP by the Popeye domain has been observed, albeit with an approximately 40-fold lower affinity compared to cAMP. A series of charge to alanine mutagenesis experiments confirmed the importance of the ultra-conserved DSPE/FQVT motifs in cAMP binding [14]. For example, mutation of aspartate 200 to alanine (D200A) in POPDC1 showed a 90% loss of cAMP affinity. A corresponding mutation of aspartate 184 to alanine (D184A) in POPDC2 also resulted in severe loss of cAMP binding [14]. The importance of the DSPE/FQVT motifs for cAMP binding was further corroborated by the discovery of a serine 201 to phenylalanine mutation (S201F) in patients suffering from limb-girdle muscular dystrophy and AV-block [29]. The resulting POPDC1^S210F^ mutant protein displayed a 50% reduction in cAMP binding. 

## 6. POPDC Proteins and Cardiac Arrhythmias 

Knockout mutations of *Popdc1* and *Popdc2* were generated in mice by replacing the first coding exon with a LacZ reporter gene [11,12,14]. No embryonic lethality was found in either of the homozygous mutants. LacZ staining for *Popdc1* and *Popdc2* revealed an exclusive expression in cardiac myocytes with an overlapping expression pattern of *Popdc1* and *Popdc2* [11,12,14]. *Popdc1* is expressed at higher levels in atrial than ventricular myocytes in both chicken and mouse hearts [4,11,14,98] while *Popdc2* on the other hand is homogeneously expressed at equal levels in all heart chambers. Similar isoform-specific differences in the expression pattern were also reported for the human heart [7]. There is significantly higher expression of both *Popdc1* and *Popdc2* in the CCS of the mouse heart, which prompted an investigation of cardiac pacemaking and conduction by electrocardiography radiotelemetry in conscious animals [14]. Subjecting *Popdc1* and *Popdc2* null mutants to physical or emotional stress or isoproterenol injection induced a sinus node bradycardia that was absent at baseline. Both mutants developed extensive sinus pauses, and showed episodes of tachybradycardia, an increased heart rate variability, and an overall reduced mean heart rate [14]. The phenotype developed in an age-dependent manner in homozygotes, while heterozygous mutants were indistinguishable from wild type animals. 

Apart from sinus bradycardia, analysis of the SAN morphology and histology revealed significant structural alterations in both mutants [14]. SAN pacemaker cells have long, thin cell protrusions which warranted them the name spider or spindle and elongated spindle cells [99]. These cells are typically electrically poorly coupled and represent a primitive embryonic-like form of cardiac myocytes that are embedded in a thick mesh of extracellular matrix and thereby are electrically isolated, preventing them from getting hyperpolarized by the much larger mass of atrial chamber myocytes [100]. However, whole-mount immunohistochemical preparations, using HCN4 as an SAN marker, revealed that in *Popdc1* and *Popdc2* null mutants there were fewer spindle cells, a reduced number of cellular extensions in the remaining pacemaker myocytes, and an SAN structure that was more compact than in its wild type counterpart [14]. These findings are significant in that a lack of cellular extensions on spindle cells may lead to an impaired electrical conduction from the SAN to the surrounding atrial cardiomyocytes. Moreover, the loss of nodal pacemaker myocytes could become a limiting factor for cardiac pacemaking under stress. The pathological phenotype and structural changes of the SAN are not present in young mice but were found in *Popdc1* and *Popdc2* null mutants that were five months and older [14]. At this stage, it is unclear whether the electrical changes are the consequence of the structural changes or vice versa. 

Similar to the cardiac arrhythmia phenotype observed in mice, *popdc1* and *popdc2* zebrafish morphants and the *popdc1^S191F^* KI mutant also presented a cardiac arrhythmia phenotype, albeit in form of an AV-block as opposed to the stress-induced sinus bradycardia that was recorded in mice [29,101]. The cardiac arrhythmia was already present at the embryonic stage and the severity of the phenotype increased in an age-dependent manner. Loss-of-function experiments showed that zebrafish larvae of three-to-four days post fertilization experienced AV-block type I, whilst larvae of older age developed complete heart block or occasionally a non-contracting heart [29,101].

Morpholino-mediated knockdown of *popdc1* and *popdc2* also induced a severe muscular dystrophy phenotype, which was characterized by an impaired formation of the myotendinous junction (MTJ), which probably was the reason for the myofiber ruptures which were frequently present in the morphants [29,101]. The MTJ is a specialized basement membrane present in skeletal muscle and is required for proper force transmission between the tendon and the muscle. It is a complex structure formed through the interaction of various extracellular matrix proteins and several membrane proteins [102,103]. Electron microscopy of the *popdc1^S191F^* KI mutant revealed a lack of the electron-dense matrix proteins within the MTJ [29,101]. These findings collectively suggest that POPDC proteins play an important role in MTJ formation. A possible hint towards the discovery of the molecular pathway affected by loss of *popdc1* and *popdc2* is drawn from the recent discovery that POPDC1 interacts with dystrophin, which plays an important role in MTJ formation in zebrafish [29,101].

SAN dysfunction in *Popdc1* and *Popdc2* null mutants is reminiscent of sick sinus syndrome (SSS) in patients. SSS is the most frequent reason for pacemaker implantation in the elderly in the absence of any other heart disease [104]. Therefore, it was initially hypothesized that POPDC proteins might have a modulatory function in the pacemaker current I_f_ [105]. To test this hypothesis, the I_f_ current density and activation time were measured in SAN myocytes that were isolated from *Popdc2* null mutant and wild-type mice [14]. Recordings that were taken at basal conditions and after stimulation with the cAMP analogue 8-Br-cAMP showed no difference between genotypes. Therefore, it is likely that other proteins involved in cardiac pacemaking are regulated in their activity by POPDC proteins.

As outlined above, in SAN myocytes, I_f_ is an important current for pacemaking that is uniquely generated by HCN4. However, the oscillating pacemaker potential is produced through a collaboration of several sarcolemmal ion channels and pumps collectively called the membrane clock, of which one or more could possibly be modulated through interaction with POPDC proteins. For example, the sodium calcium exchanger NCX1 was recently identified as a POPDC2 interacting partner and the loss of NCX1 was shown to cause SAN dysfunction with a phenotype similar to the one observed in POPDC mutants [106,107]. NCX1 is part of the membrane clock and functionally couples the membrane clock to the Ca^2+^ clock [108]. 

TWIK-related K+ channel 1 (TREK-1) or KCNK is a background potassium channel, the main role of which, in the heart, is to control cell excitability and maintain the membrane potential below the threshold of depolarization [109]. It was one of the first POPDC-interacting proteins to be identified in the heart. The cardiac-specific knockout of *Kcnk2,* which encodes TREK-1, produces a stress-induced sinus bradycardia with a phenotypical manifestation that largely resembles the one described for *Popdc1* and *Popdc2* null mutants [110]. Therefore, a hypothesis was put forward suggesting that an aberrant TREK-1 current causes the sinus bradycardia in POPDC null mutants. Co-expression of TREK-1 and the three POPDC isoforms in *Xenopus* oocytes revealed a two-fold higher TREK-1 current and is probably the result of an increased membrane trafficking of TREK-1 in the presence of either POPDC isoform [14]. When cAMP levels were raised in frog oocytes, the stimulatory effect of POPDC proteins on TREK-1 current was lost [14]. It is therefore plausible that POPDC proteins modulate membrane trafficking of TREK-1, whilst cAMP, by binding to POPDC, regulates its interaction with TREK-1. The interaction of POPDC proteins with TREK-1 has been mapped to the Popeye domain by deletion analysis and based on that knowledge, a bi-molecular Förster-resonance energy transfer (FRET) sensor was constructed [14,29]. The FRET ratio obtained at baseline decreased after the addition of isoproterenol or FSK, confirming that cyclic nucleotide binding affects the interaction of POPDC1 with TREK-1 [14]. These findings give support to the hypothesis that an aberrant TREK-1 current causes sinus bradycardia in *Popdc1* or -*2* null mutants. Loss of POPDC proteins at the plasma membrane should lead to a reduction in TREK-1 current, which in turn should make the cell more excitable, however, the opposite is true for the *Popdc1* and *-2* null mutants [14]. However, one must be careful to extrapolate from *Xenopus* oocytes to SAN pacemaker myocytes. There may be cell-type specific differences in the regulation of membrane trafficking or protein–protein interaction and thus a direct measurement of TREK-1 current in SAN pacemaker cells is required to rule out its functional involvement in the sinus bradycardia phenotype. It is likely that POPDC proteins are part of a complex network of proteins involved in modulating sinus node pacemaking at baseline and after ANS stimulation. Therefore, we currently favor the view that the pacemaker phenotype of POPDC null mutants is probably the result of many different feedback regulations and unlikely the result of a single aberrantly regulated ion channel. 

A recent study by Tibbo et al. (2020) showed that POPDC1 proteins, similar to other cAMP effectors, also form a complex with PDE4s, particularly and preferably with the PDE4A isoform [111]. The study concluded that POPDC1–PDE4A complex formation serves as a protective measure, which prevents inappropriate cAMP binding to the Popeye domain under basal conditions. Experimentally orchestrated disruption of PDE4 and POPDC1 interaction resulted in a decreased interaction between POPDC1 and TREK1 as well as causing a prolongation of AP duration in isolated ventricular myocytes [111]. 

While the precise molecular interactions still need to be worked out, the protein complex in which POPDC proteins are probably working to modulate action potential duration is gradually emerging. TREK-1 binds to the proximal part of the Popeye domain and binding may be adjacent or overlapping with that of PDE4 [29,111]. Thus, probably, these three proteins are in a common complex. TREK-1 also interacts with the AKAP protein AKAP79/150 and is subject to PKA-dependent phosphorylation, resulting in inactivation of TREK-1 current [112,113,114]. Thus, POPDC1 could be involved in balancing and fine-tuning the effect of βAR receptor stimulation on TREK-1 current. However, further work is required to identify the relevant molecular and functional interactions and address the question of whether POPDC proteins are part of the AKAP79/150-PKA complex or are located adjacent to it.

The interaction and complex formation between POPDC1 and PDE4A may also be relevant for the altered hippocampal synaptic plasticity that was recently described for *Popdc1* null mutants [115]. Activity-dependent modulation of synaptic plasticity is essential to learning and memory, and its many forms are associated with a complex set of molecular signaling pathways [116]. The cAMP–PKA-mediated pathways are of particular importance in persistent forms of synaptic plasticity characterized by long-term potentiation (LTP) or long-term depression (LTD) [117]. PKA activation functions like a gate between transient and persistent forms of LTP [117,118]. POPDC1 is found in different subregions of the hippocampus including CA1 and seems to be particularly enriched in the synaptic membrane fraction, which supports a potential role in modulating synaptic plasticity [115]. Indeed, high-frequency electrical stimulation of hippocampal slices isolated from *Popdc1* null mutants showed enhanced LTP, especially in response to a single-train high-frequency electrical stimulation, which suggests that loss of *Popdc1* probably reduces the threshold for LTP induction [115]. Pharmacological treatment of hippocampal slices of *Popdc1* null mutants with FSK or FSK and IBMX (an unspecific PDE antagonist) differed in their effects on LTP formation. Enhancement of the LTP response was observed in *Popdc1* null mutant samples that were treated with FSK while FSK/IBMX treatment caused a decrease in LTP formation. It is currently unclear why these treatments triggered a different outcome; however, it can be hypothesized that in the absence of *Popdc1*, the large surge in cAMP in response to FSK/IBMX treatment triggers a negative feedback loop, which might cause a reduction in the LTP response. Therefore, these results can be interpreted as evidence for a buffering function of POPDC proteins to prevent abnormal levels of cAMP accumulation in cells and thereby ensuring a graded response to different cAMP levels. PKA inhibition blocks enhanced LTP formation in *Popdc1* null mutants, which suggests that POPDC proteins similar to its function in cardiac myocytes are probably involved in fine tuning and limiting LTP formation by preventing PKA-driven phosphorylation of target proteins in response to subthreshold LTP-inducing stimuli [115]. 

## 7. POPDC Proteins and Plasma Membrane Compartments

Specialized membrane structures called caveolae form segregated pockets in the plasma membrane to create membrane microdomains, which are involved in the precise regulation of and crosstalk between signaling pathways [119]. Caveolae are rich in cholesterol and glycosphingolipids and are structurally stabilized by scaffolding proteins called caveolins (Cav1–Cav3). Cav-3 is the muscle-specific isoform, which localizes to the sarcolemma in skeletal muscle fibers and in the plasma membrane and t-tubules of cardiac myocytes [120]. Caveolae are at the center of various processes such as vesicular trafficking, mechanosensation and transduction, as well as signaling processes such as βAR signaling. A number of different ion channels and transporters have been localized to caveolae in cardiac myocytes including the L-type Ca^2+^ channel (LTCC), SCN5A, and HCN4 [121]. POPDC1 has also been shown to localize within the caveolae compartments where it binds Cav3 via a consensus sequence found in the carboxy-terminal end of the Popeye domain [24]. The presence of POPDC1 in caveolae was suggested to be critical for cardiac protection and ischemic preconditioning [24]. In the absence of *Popdc1*, caveolae were found to be reduced in number and increased in size in cardiac myocytes, which probably contributes to the observed ischemia/reperfusion vulnerability of *Popdc1* null mutant hearts [24]. Acute ischemic injury using mouse hearts subjected to retrograde Langendorff perfusion revealed that *Popdc1* null mutant hearts develop larger infarcts and significantly reduced functional recovery [24]. 

Impaired calcium transients were also reported in cardiac myocytes isolated from *Popdc1* null mutants, which may partially explain their cardiac arrhythmia phenotypes [24]. In cardiac myocytes, Cav3 interacts with the L-type calcium channel Ca_V_1.2, which results in the formation of a cAMP-signaling compartment that consist of Ca_V_1.2, the β_2_-AR, PKA, and AC [122]. It is therefore possible that POPDC proteins, being an interaction partner of Cav3, are also part of this signaling complex. In ventricular cardiac myocytes of rat, mouse, human, and canine origin, both POPDC1 and POPDC2 are found at the intercalated disk (ID) [46,123]. The ID is a specialized subcellular region of cardiac myocytes and important for their electrical and mechanical coupling [124]. Three different macromolecular complexes are found in IDs: the fascia adherens which function as actin anchoring sites; gap junctions, which are involved in electrical coupling; and desmosomes and adherens junctions, which are important for mechanical connection of cardiac myocytes [124]. Recently, an interaction of XIRP1 and POPDC1 and POPDC2 has been reported [46]. XIRP1 is a scaffolding protein which modulates the integrity of the ID. XIRP1 interacts with β-catenin and p120-catenin and through these protein–protein interactions it associates with N-cadherin in the adherens junction [125]. Likewise, XIRP1 modulates gap-junction function through the modulation of connexin 43 phosphorylation [126]. Moreover, missense mutations in *XIRP1* have been associated with Brugada syndrome and nocturnal sudden cardiac death [127]. Thus, there is a significant functional overlap between the two proteins. Nevertheless, whether POPDC modulation of XIRP1 function is controlled via cAMP signaling is currently unknown. The same study that reported the XIRP1 interaction also demonstrated an interaction of POPDC1 with annexin A5 (ANXA5) [46]. Together with other annexin subunits, ANXA5 is recruited to the injured plasma membrane and forms a 2-dimensional array preventing an enlargement of the damaged area [128]. Furthermore, Cav3 and dysferlin have been linked to plasma-membrane repair in striated muscle and are interacting with POPDC proteins [24,29,129,130,131]. A recent report identified POPDC1 as a novel interactor of anoctamin 5 (ANO5) [132]. ANO5 has also been linked to plasma membrane repair in skeletal muscle [133]. Thus, several proteins involved in plasma membrane repair in skeletal muscle have now been shown to be interacting with POPDC1. However, thus far, no direct test has been performed to demonstrate an involvement of POPDC1 in plasma-membrane repair. However, membrane discontinuities have been reported in skeletal muscle biopsies of patients carrying a POPDC1^S201F^ mutation [29]. Additionally, many patients carrying POPDC mutations show high serum creatinine kinase levels (Table 1), which suggests that, possibly, the sarcolemma of patients carrying POPDC mutations is leaky, potentially due to an impaired plasma membrane repair. 

## 8. Mutations in POPDC Genes Are Causing Heart and Muscle Disease

Recently, several patients carrying mutations in POPDC genes have been identified. To date, seven mutations in *POPDC1*, two in *POPDC2*, and three in *POPDC3* have been described (Table 1). Commonly, these patients suffer from skeletal muscle and heart disease. Patients carrying mutations in *POPDC1* develop a recessive form of limb-girdle muscular dystrophy (LGMDR25) with highly variable onset and in addition displaying sinus bradycardia and an AV-block of varying severity [29,134,135,136,137]. Mutations found in *POPDC2* cause patients to develop a cardiac arrhythmia phenotype but importantly do not display any skeletal muscle phenotype [138,139]. In contrast, patients carrying mutations in *POPDC3* display a severe limb-girdle muscular dystrophy (LGMDR26) but a normal heart [140]. The differential tissue tropism of the different POPDC gene mutations is likely due to their postnatal expression pattern, whereby *POPDC1* is expressed relatively evenly in both muscle and heart, whereas *POPDC2* is predominantly expressed in the heart, while *POPDC3* expression is mostly found in skeletal muscle [4].

Their phenotypic presentation varies, ranging from a mild AV block to a complete heart block which can be either nocturnal or persistent. Their common symptoms are muscle weakness and elevated serum creatine kinase (CK) levels [29,134,135,136,137]. Immunohistochemical staining of muscle biopsies from patients with either of the *POPDC1* mutations uniformly revealed a loss of membrane localization of mutant as well as of POPDC2 proteins [29,134,135,136,137]. These findings suggest that the underlying cellular pathology of *POPDC1* mutations in these patients is probably an aberrant membrane trafficking of the mutant protein. Mutations in *POPDC3* genes have also been linked to LGMD, however, contrary to the *POPDC1* mutations, analysis of patient biopsies did not show any aberrant membrane localization of POPDC1, POPDC2, or mutant POPDC3 proteins [140].

*POPDC1^S201F^* is the first mutation that was discovered in patients [29]. This recessive mutation affects serine 201, which is a residue of the invariable DSPE motif of the PBC (Figure 2). It is thought that the substitution of the small serine to a much bulkier phenylalanine interferes with ability of cAMP to bind to the PBC. Indeed, when the cAMP-binding capacity of this mutant protein was compared to wild type POPDC1, a 50% reduction in binding affinity was noted [29]. *POPDC1^S201F^* also caused higher TREK-1 current levels when expressed in *Xenopus* oocytes despite a decreased membrane transport of the mutant channel. The higher current is probably generated because TREK-1 in a complex with POPDC1 is protected from getting inactivated by PKA-dependent phosphorylation [29].

Clinically, *POPDC1^S201F^* patients, who are a three-generation family of Albanian descent carried the mutation to homozygosity. The eldest patient developed a late onset LGMD in his 40s and lost his ambulation in his 60s. Two younger patients suffered from syncopal episodes that began in adolescence. These patients also showed a type II AV block. However, only one of the younger patients developed sinus bradycardia. Despite elevated CK values, no muscular dystrophy could be identified in the younger patients, suggesting that LGMD is a late onset feature of the disease [29]. However, subsequent reports of different mutations revealed a wide spectrum of early and late onset of skeletal muscle disease and most patients developed a limb-girdle muscular dystrophy [29,134,135,136,137]. LGMD is a highly diverse group of muscular dystrophies, which have been linked to a wide variety of proteins affecting glycosylation of dystroglycan, are involved in mechanical signaling (sarcoglycans) and in mitochondrial function [141]. Three genes, *DYSF*, *ANO5* and *CAV3* have been linked to LGMD and encode proteins, which have been found to interact with POPDC proteins [24,29,132]. One patient, who carries a POPDC1 mutation, developed an Emery–Dreifuss muscular dystrophy (EDMD). EDMD typically is caused by proteins that are part of the nuclear envelope associated with contractures of the joints. EDMD is otherwise phenotypically similar to LGMD including the presence of cardiac arrhythmia phenotypes in both diseases [142]. As noted previously, a nuclear envelope localization of POPDC proteins has been reported [35,36]. 

Furthermore, POPDC1 proteins have recently been implicated in long QT syndrome. Many disease-associated single-nucleotide polymorphisms (SNPs) reside preferentially in enhancer elements that regulate gene expression. With this in mind, Wang and colleagues applied a new approach to analyze data from genome wide association studies (GWAS) for biologically relevant sub-threshold variants in loci that were previously missed [143]. In their study, they focused on QT intervals and QRS duration during cardiac conduction. The authors discovered a novel locus that affects a cardiac enhancer that controls the expression of *POPDC1* and *POPDC3,* whereby the associated SNP impairs nuclear factor I binding [143]. Another GWAS study looking at SNPs that prolong the QT interval in Hispanics/Latinos also found linkage to *POPDC1* [144]. Currently, there is no evidence for a prolongation of the QRS complex in the mouse, while in zebrafish *popdc1* morphants, a reduction in action potential duration has been described [143]. Significantly, action potential prolongation was observed in rabbit ventricular myocytes when the interaction of PDE4A and POPDC1 was blocked by a peptide [111]. These findings indicate that it is probably important to choose the right animal model to find evidence for a functional link between *POPDC1* mutations and QT interval and QRS duration. 

A group of patients that carry a heterozygous nonsense mutation in *POPDC2* were recently identified [138]. In these patients, the POPDC2 protein is truncated due to a premature stop codon at position 188 (POPDC2^W188X^), which causes a partial loss of the functionally important Popeye domain. Surprisingly, it was shown that POPDC2^W188X^ retained its ability to bind cAMP with similar affinity as its wild-type counterpart as well as to interact with the two-pore potassium channel TREK-1 [138]. However, co-expression experiments of POPDC2^W188X^ and TREK-1 in *Xenopus* oocytes revealed that the mutant protein produces an abnormal current modulation, suggesting it to represent a pathogenic mutation. A knock-in mutant was generated in mice and, similar to the *Popdc2* null mutant, the *Popdc2^W188X^* develops a stress-induced sinus bradycardia phenotype [138]. Thus, it is apparent that the mouse probably mainly manifests its cardiac arrhythmia phenotype as sinus node dysfunction whereas in zebrafish and patients, AV conduction abnormalities are observed. These species-specific differences may be related to the vastly different heart rates of the mouse compared to zebrafish and man, or due to differences in the utilization of ion channels for action potential generation. Patients carrying the POPDC2^W188X^ only develop a cardiac phenotype, while skeletal muscle appears unaffected. It cannot be ruled out that there is a late onset of skeletal muscle pathologies in case of *POPDC2* mutations. However, we favor the interpretation that heart-specific effects of POPDC2 mutations are probably due to its preferential expression in the heart. This view is corroborated by the fact that a complementary phenotype is observed for mutations in *POPDC3*, which mainly affects skeletal muscle, while the heart is normal [140].

A novel missense POPDC2^L245P^ mutation has been recently reported in a family of four that developed congenital junctional ectopic tachycardia (cJET) [139]. cJET is an extremely rare tachyarrhythmia which develops in infants, and previously, only a single gene, *TNNI3K*, has been linked to familial cJET cases [145]. Abnormal automaticity of the AV node has been described as the primary pathophysiologic mechanism underlying cJET. The cJET pathology appears to represent a gain-of-function phenotype and appears to represent the opposite of the AV-block that has been seen in case of the POPDC2^W188X^. It also should be pointed out that, in contrast to *POPDC1* and *POPDC3* mutations, which both are recessive and only the homozygous patient develops a pathology, in the case of *POPDC2* both known mutations display a dominant trait. 

## 9. Concluding Remarks

The Popeye domain-containing genes have now been known for more than 20 years [4,5]. The association of POPDC gene mutations with striated muscle disease, however, has only recently (2016) been established. Model organisms, which carry loss-of-function or missense mutations found in patients, have been developed. The phenotypes presented in these animal models largely overlap with the pathologies found in patients carrying POPDC mutations. This is encouraging, as it reinforces these model organisms as being suitable to work out the underlying pathogenic mechanism of human POPDC mutations. Additionally, the overlapping nature of the aforementioned phenotypes suggests that POPDC proteins play a fundamental and essential role in cardiac electrophysiology. Recent work defining the role of POPDC1 in hippocampal long-term potentiation suggests that POPDC proteins’ role in membrane biology is not confined to the heart [115]. It is possible that the underlying molecular pathways in which POPDC proteins are involved in hippocampal neurons and cardiac myocytes may be similar or even identical. Therefore, it is possible, that the enhanced LTP and the stress-induced sinus bradycardia found in *Popdc1* null mutant mice may be based on the same or related molecular defects. However, these molecular defects are currently not fully understood and need to be investigated further. 

The role of POPDC proteins as an important cAMP effector has been further confirmed by the findings that show complex formation between POPDC and PDE4 proteins. This interaction is essential and interference with the PDE4–POPDC complex formation has a direct, aberrant effect on cardiac action potential. All cAMP effector proteins are either directly associated with phosphodiesterases or both proteins are part of a complex. The PDE–effector protein interaction is essential for limiting cAMP effector protein activation by cAMP. This is a principle that also applies to POPDC proteins. Therefore, an important question for the near future is: how far-reaching is the analogy? That is, do other elements of the cAMP pathway also undergo complex formation with POPDC proteins? For example, do POPDC proteins interact with other effector proteins such as EPAC or PKA? Do POPDC proteins form a complex with AKAP proteins or adenylyl cyclases? Knowledge gained in this regard will lead to a better understanding of the role that POPDC proteins play in cAMP signaling.

POPDC proteins have a cAMP-binding domain which diverges from those found in other cAMP effector proteins. Nevertheless, compelling evidence has already been produced for a direct involvement of POPDC proteins in cAMP signaling, both through ligand binding and protein–protein interaction with elements of the pathway (such as PDE4). However, because the Popeye domain is larger than the typical cAMP-binding domain, it cannot be ruled out that POPDC proteins might also participate in other signaling pathways. In particular, a link to Ca^2+^ or CaMKII signaling, which are both essential for cardiac pacemaking, cannot be ruled out [83,146,147]. In this regard an indirect involvement of POPDC proteins being, for example, a phosphorylation target by kinases after β-adrenergic stimulation, also needs to be considered [41]. Moreover, it is likely that POPDC proteins participate in the crosstalk between signaling pathways and the Ca^2+^ and cAMP-signaling pathways would be of particular interest in this regard.

Another important question that needs to be addressed in the future is whether POPDC proteins also have a structural function in the heart. They have a structural role in skeletal muscle and in epithelial cells, however this is less clear in the heart. Apart from some alterations in the structure of the sinus node cells, no structural defect has been described in POPDC mutants. However, POPDC proteins have a prominent expression in the intercalated disc (ID) and also interact with several proteins such as ZO1 [43] or XIRP1 [46], which suggests that they might possibly also have a role in controlling structure and function of the ID. From these concluding remarks, it becomes apparent that despite all the progress made and knowledge accumulated in the past 20 years, still more work on POPDC proteins needs to be conducted to better define their molecular functions in the heart and beyond.

## Figures and Tables

**Table 1:** POPDC gene mutations discovered in patients suffering from heart and skeletal muscle disease.

Gene	Mutation	Trait	Phenotype	Ref.
*POPDC1*	1A>G	rec.	AV-block, Myalgia, hCK ^1^	[134]
	R88X	rec.	AV-block, LGMD ^2^, hCK	[134]
	D92G	rec.	EDMD ^3^	[135]
	R143X	rec.	AV-block, LGMD, hCK	[136]
	S201F ^4^	rec.	AV-block, LGMD, hCK	[29]
	Del56V217-K272	rec.	AV-block, LGMD, hCK	[134]
	S263X	rec.	AV-block, LGMD, hCK	[137]
*POPDC2*	W188X	dom.	AV-block	[138]
	L245P	dom.	cJET ^5^	[139]
*POPDC3*	L155H	rec.	LGMD	[140]
	L217F	rec.	LGMD	[140]
	R261Q	rec.	LGMD	[140]

^1^ hCK: high-serum creatine kinase level. ^2^ LGMD: limb-girdle muscular dystrophy. ^3^ EDMD: Emery–Dreifuss muscular dystrophy. ^4^ POPDC1^S202F^ affects a residue of the PBC [29]. ^5^ cJET: congenital junctional ectopic tachycardia.
